# Transcriptomic reappraisal identifies *MGLL* overexpression as an unfavorable prognosticator in primary gastrointestinal stromal tumors

**DOI:** 10.18632/oncotarget.10304

**Published:** 2016-06-27

**Authors:** Chien-Feng Li, I-Chieh Chuang, Ting-Ting Liu, Ko-Chin Chen, Yen-Yang Chen, Fu-Min Fang, Shau-Hsuan Li, Tzu-Ju Chen, Shih-Chen Yu, Jui Lan, Hsuan-Ying Huang

**Affiliations:** ^1^ Department of Pathology, Chi-Mei Medical Center, Tainan, Taiwan; ^2^ National Institute of Cancer Research, National Health Research Institutes, Tainan, Taiwan; ^3^ Department of Biotechnology, Southern Taiwan University of Science and Technology, Tainan, Taiwan; ^4^ Department of Pathology, Kaohsiung Chang Gung Memorial Hospital and Chang Gung University College of Medicine, Kaohsiung, Taiwan; ^5^ Department of Pathology, Changhua Christian Hospital, Changhua, Taiwan; ^6^ Division of Oncology, Department of Internal Medicine, Kaohsiung Chang Gung Memorial Hospital and Chang Gung University College of Medicine, Kaohsiung, Taiwan; ^7^ Department of Radiation Oncology, Kaohsiung Chang Gung Memorial Hospital and Chang Gung University College of Medicine, Kaohsiung, Taiwan; ^8^ Bone and Soft Tissue Study Group, Taiwan Society of Pathology, Taiwan

**Keywords:** cancer metabolism, lipid metabolism, MGLL, transcriptome, GIST

## Abstract

The role of deregulated cellular metabolism, particularly lipid metabolism, in gastrointestinal stromal tumors (GISTs) remains unclear. Through data mining of published transcriptomes, we examined lipid metabolism-regulating drivers differentially upregulated in high-risk cases and identified monoglyceride lipase (*MGLL*) as the top-ranking candidate involved in GIST progression. *MGLL* expression status was examined in three GIST cell lines and two independent sets of primary localized GISTs. *MGLL* mRNA abundance and immunoexpression was determined in 70 cases through the QuantiGene assay and H-scoring on whole sections, respectively. H-scoring was extended to another cohort for evaluating MGLL immunoexpression on tissue microarrays, yielding 350 informative cases, with *KIT/PDGFRA* mutation genotypes noted in 213 of them. Both imatinib-sensitive (GIST882) and -resistant (GIST48 and GIST430) cell lines exhibited increased MGLL expression. *MGLL* mRNA levels significantly increased from adjacent normal tissue to the non-high-risk group (*p* = 0.030) and from the non-high-risk group to high-risk GISTs (*p* = 0.012), and were associated with immunoexpression levels (*p* < 0.001, r = 0.536). *MGLL* overexpression was associated with the nongastric location (*p* = 0.022) and increased size (*p* = 0.017), and was strongly related to mitosis and risk levels defined by NIH and NCCN criteria (all *p* ≤ 0.001). Univariately, *MGLL* overexpression was strongly predictive of poorer disease-free and overall survival (both *p* < 0.001), which remained prognostically independent for both endpoints, along with higher risk levels. Conclusively, MGLL is a lipid metabolic enzyme causatively implicated in GIST progression given its association with unfavorable clincopathological factors and independent negative prognostic effects.

## INTRODUCTION

Gastrointestinal stromal tumors (GISTs) potentially originate from interstitial Cajal cells or their precursors. They are usually genetically characterized through mutually exclusive gain-of-function mutations of KIT protooncogene receptor tyrosine kinase (*KIT*) or platelet-derived growth factor receptor alpha (*PDGFRA*) genes [1]. *KIT* and *PDGFRA* mutations elicit constitutive activation of the corresponding receptor tyrosine kinases, which drive tumor inception and dictate treatment response to imatinib [1-4]. The *KIT/PDGFRA* genotypes are variably associated with the aggressiveness of resected imatinib-naïve GISTs [3-5]; however, their prognostic value has not been uniformly validated in previous studies [6-8]. Although both the National Institutes of Health (NIH) and National Comprehensive Cancer Network (NCCN) risk schemes are prognostically useful [9, 10], more accurate prognostication is becoming a critical issue in the postimatinib era for counseling regarding outcomes and for the identification of targetable aberrant molecules other than receptor tyrosine kinases. Therefore, identifying candidate deregulated molecules of other signaling pathways is essential for resolving the current limitations in prognostication and therapy.

Compared with normal cells, cancer cells voraciously consume nutrients, including glucose, lipids, and amino acids, through unique signaling pathways; molecular aberrations in metabolism-associated enzymes may alter these pathways [11]. Despite being a cancer hallmark of renewed interest, extremely little has been clarified regarding the pathogenetic, biological, and clinical relevance of metabolic reprogramming in mesenchymal neoplasms, including GISTs [11]. Of the deregulated metabolic pathways, the de novo biosynthesis of lipids is drastically increased in rapidly proliferating cancer cells because lipids provide the building blocks of lipid rafts and various protumorigenic lipid-signaling molecules and engage in coordinating cell motility and signaling cascades [12-14]. However, the clinical and biological relevance of the lipolytic pathway, required for an immediate liberation of reserved fatty acids for metabolic and signaling demands in cancer cells, warrants further exploration [14]. Thus, considering lipid metabolic processes, we began reappraising the published transcriptome of GISTs to search for metabolic driver genes differentially expressed between high-risk and non-high-risk cases. A top-ranking upregulated gene identified through this data-mining approach was the monoglyceride lipase-encoding *MGLL* (also called *MAGL*, encoding monoacylglycerol lipase), a serine hydrolase that preferentially hydrolyzes monoglycerides into fatty acids and glycerol [14, 15].

Thus far, no study has directly evaluated the implications of altered *MGLL* expression in GISTs. By using two independent cohorts (Supplementary Figure 1), we validated the clinical relevance of *MGLL* overexpression to the full extent of transcriptional and translational characterization and clearly demonstrated that *MGLL* overexpression is strongly correlated with its mRNA and protein levels and adverse clinicopathological factors, and has independent prognostic utility in identifying aggressive cases. Regarding deregulated lipid metabolism, our findings provide further prognostic information as well as biological insights into the pathways regulating GIST progression.

## RESULTS

### Differentially upregulated *MGLL* expression in high-risk GISTs

**Figure 1:**
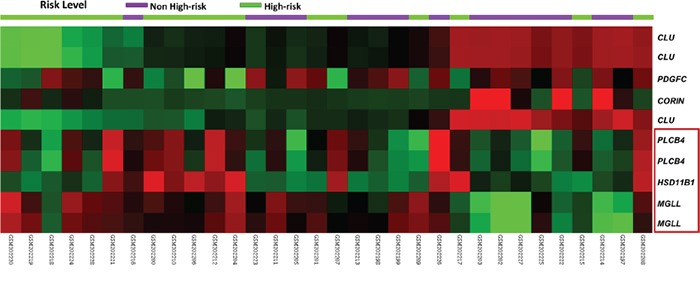
Heatmap of unsupervised hierarchical clustering analysis for differentially expressed genes associated with lipid metabolism Two roughly segregated clusters were identified, which comprised more high-risk (*green*) GISTs on the left and more non-high-risk (*purple*) GISTs on the right. *HSD11B1* (hydroxysteroid (11-beta) dehydrogenase 1), *MGLL* (monoglyceride lipase), and *PLCB4* (phospholipase C beta 4) genes were top-ranking candidates highlighted in a red bracket, of which *MGLL* were selected for validation in this study. According to their fold changes, increased and decreased expression levels of individual genes were expressed in red and green of varying intensity, respectively.

Considering 559 probes covering 274 genes regulating the lipid metabolic process, we conducted unsupervised clustering on the transcriptomic dataset of 32 GISTs (GSE8167). This approach crudely segregated the samples into two clusters (Figure 1), with several notable genes differentially expressed between the high-risk and non-high-risk cases. Of these, *HSD11B1*, *MGLL*, and *PLCB4* were the three top-ranking upregulated candidates exhibiting remarkable expression fold changes (log_2_ ratio >1 or <−1) and strong associations with the high-risk category (all *p* ≤ 0.0001, Supplementary Table 2). Given the novel discovery of the involvement of MGLL-driven lipolysis in promoting carcinogenesis through the modulation of the fatty acid network [14, 15], we further validated the clinical relevance and prognostic implication of *MGLL* in cell lines and two independent tumor cohorts.

### *MGLL* mRNA abundance is positively associated with risk levels and protein expression

**Figure 2:**
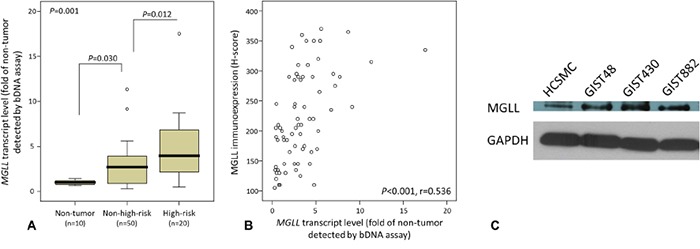
MGLL (monoglyceride lipase) mRNA upregulation and protein overexpression and their strong correlation validated in GIST samples and cell lines **A.** Compared with the normal tissues, *MGLL* mRNA abundance was determined to be differentially upregulated across GISTs of various risk levels (informative n=70) and exhibit significant risk level-associated increment, indicating its role in tumor progression. **B.** In the same set of 70 GISTs, the scattered plot demonstrated a strong correlation between the levels of *MGLL* mRNA measured by Quantigene assay on the *X* axis and H-scores of MGLL protein immunoexpression on the *Y* axis (see representative images in Figure 3). **C.** Western blotting assay revealed increased endogenous expression of MGLL protein in all imatinib-resistant (GIST48, GIST430) and imatinib-sensitive (GIST882) cell lines, compared with the reference human colonic smooth muscle cells (HCSMC). GAPDH (glyceraldehyde-3-phosphate dehydrogenase) was used as the loading control.

Adopting the same comparative logic in transcriptomic reappraisal, we preselected all 86 GISTs (cohort 1 in Supplementary Figure 1) for *MGLL* mRNA quantification to ensure congruity in the assignment of high-risk versus non-high-risk categories by using both the NCCN and NIH schemes [9, 10]. As an indicator of mRNA abundance, the luminescence-based detection of dioxetane alkaline phosphatase substrate was informative in 10 normal tissue samples and 70 primary localized tumors, whereas another 16 tumors could not be analyzed because of nucleic acid degradation. These 70 informative GISTs, 49 gastric and 21 intestinal, were classified as high-risk in 20 cases and non-high-risk in 50 (Supplementary Table 1). Compared with normal tissues, *MGLL* mRNA abundance was significantly higher in the GISTs in whole sections (*p* = 0.001, Figure 2A) and increased from normal tissues over the non-high-risk group (*p* = 0.030) to the high-risk group (*p* = 0.012), confirming the promotional role of *MGLL* in tumor progression. Notably, *MGLL* mRNA and MGLL levels were strongly and positively associated with each other (*p* < 0.001, r = 0.536; Figure 2B).

### Increased endogenous MGLL expression in GIST cell lines

We further compared the endogenous MGLL expression in GIST cell lines versus the reference human colonic smooth muscle cells (HCSMCs). Irrespective of the sensitivity or resistance to imatinib, all three GIST cell lines demonstrated increased endogenous MGLL levels in western blot analysis (Figure 2C).

### MGLL overexpression is associated with unfavorable clinicopathological factors

**Figure 3:**
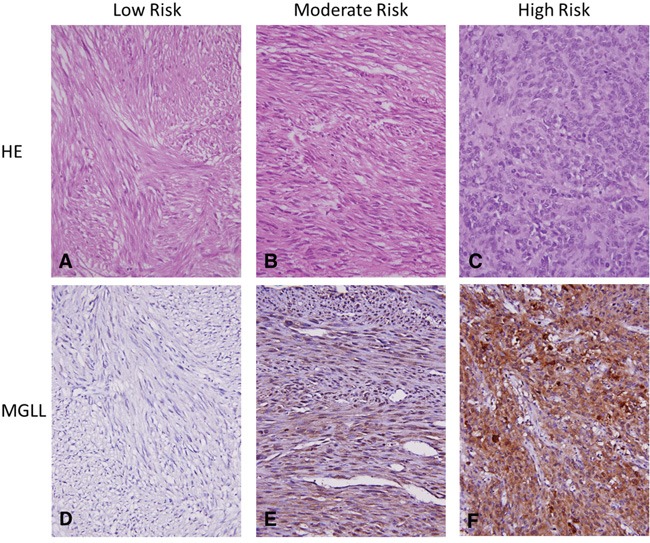
Representative histology and MGLL (monoglyceride lipase) immunoexpression in GISTs of various risk categories The hematoxylin eosin stains for histological evaluation (X400) showed gradually increased cellularity from low- **A.**, intermediate- **B.** to high-risk **C.** GISTs, which exhibited no **D.**, weak **E.**, and strong **F.** cytoplasmic immunoexpression of MGLL (monoglyceride lipase, X400), respectively.

Given the strong correlation between MGLL protein and *MGLL* mRNA expression levels, we next systematically analyzed the clinical relevance of *MGLL* immunoexpression in a large validation set of GISTs (cohort 2 in Supplementary Figure 1) through tissue microarray (TMA)-based immunohistochemistry. We included 350 informative GISTs with available follow-up data, comprising 88 no-risk or very-low-risk, 100 low-risk, 65 moderate-risk, and 97 high-risk cases defined using the NCCN scheme (Table 1) and corresponding to 127 very-low-to-low-risk, 110 intermediate-risk, and 113 high-risk cases according to the NIH scheme (Supplementary Table 3). As indicated in Table 1, *MGLL* overexpression was associated with nongastric location (*p* = 0.022) and increased size (*p* = 0.017) and strongly correlated with increased mitosis and risk levels defined by both the NIH and NCCN schemes (all *p* ≤ 0.001, Figure 3); however, the overexpression was not associated with unfavorable genotypes (*p* = 0.540).

**Table 1:** Clinicopathological and *KIT/PDGFA* genotypic correlations with MGLL immunoexpression in cohort 2 of primary GISTs in in tissue microarrays

	MGLL Expression		*p*-value
	Low	High	
**Sex**			0.915
Male	86	87	
Female	89	88	
**Age (years)**	60.14±13.026	59.59±12.527	0.749
**Location**			0.022*
Gastric	116	95	
Non-gastric	59	80	
**Histologic Type**			0.080
Spindle	140	126	
Epithelioid & Mixed	35	49	
**Tumor Size (cm)^&^**	5.895+/−4.0668	6.914+/−4.3204	0.017*
**Mitotic Count (50HPFs)^&^**	6.75+/−18.687	11.71+/−27.174	<0.001*
**NIH Risk**			0.001*
Low/Very low	73	54	
Intermediate	62	48	
High	40	73	
**NCCN Guideline**			<0.001*
None/Very low	57	31	
Low	55	45	
Moderate	32	33	
High	31	66	
**Mutation Types**			0.540
Favorable Types	51	55	
Unfavorable Types	47	60	

NIH, National Institutes of Health; NCCN, National Comprehensive Cancer Network; MGLL, monoglyceride lipase; *: Statistically significant; ^&^: Wilcoxon rank-sum test.

### *MGLL* overexpression independently predicts worse outcomes

**Figure 4:**
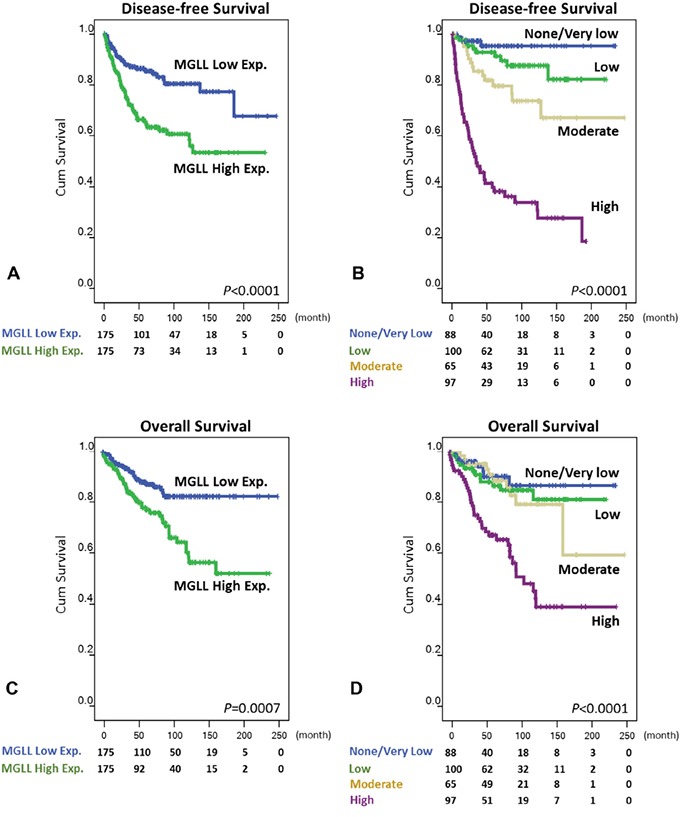
Kaplan-Meier analyses of univariate disease-free survival **A, B.** and overall survival **C, D.** in patients with primary GISTs according to MGLL (monoglyceride lipase) immunoexpression status (A, C) and risk levels defined by National Comprehensive Cancer Network scheme (B, D).

Unlike overall survival (OS), the disease-free survival (DFS) of patients receiving primary surgery is not confounded by imatinib therapy administered for relapsed and disseminated diseases. Therefore, we primarily detailed disease-free DFS-related findings in this article. Univariately, *MGLL* overexpression was strongly predictive of worse outcomes (Figure 4A, *p* < 0.0001), with the median DFS being 42.7 months in the *MGLL*-overexpressing GISTs and 61.0 months in their *MGLL*-underexpressing counterparts. The nongastric location (*p* = 0.0023) and unfavorable mutated *KIT/PDGFRA* genotypes (*p* = 0.0005) significantly predicted inferior DSS. Moreover, significant poor prognosticators of DFS included the presence of epithelioid histology, increased tumor size and mitosis, and higher GIST risk defined by both the NIH and NCCN schemes (all *p* < 0.0001; Figure 4B, Table 2, and Supplementary Table 3). Regarding OS at the univariate level (Supplementary Tables 4 and 5), age older than 70 years (*p* = 0.0048) was a significant poor prognosticator, whereas nongastric location (*p* = 0.0807) showed only a marginal trend toward poorer OS. Notably, *MGLL* overexpression (*p* = 0.0007, Figure 4C) still strongly indicated poorer OS, which was also significantly associated with increased tumor size and mitosis and higher GIST risk defined by both the NCCN and NIH schemes (Figure 4D; all *p* < 0.0001). Although epithelioid histology was a significant prognosticator (*p* = 0.002), it was not as powerful as that seen in DFS.

**Table 2:** Univariate and multivariate disease-free survival analyses according to MGLL expression status, NCCN guidelines, and other prognostic factors

Parameter	Univariate analysis			Multivariate analysis		
	No. Case	No. Event	*p*-value	HR	95% CI	*p*-value
**Sex**			0.4667			
Male	177	43				
Female	173	44				
**Age (years)**			0.0584			
<70	259	59				
>=70	91	28				
**Location**			0.0023*			0.875
Gastric	211	40		1	-	
Non-gastric	139	47		0.961	0.585-1.578	
**Histologic Type**			<0.0001*			0.001*
Spindle	266	51		1	-	
Mixed/Epithelioid	84	36		2.266	1.374-3.736	
**Tumor Size (cm)^#^**			<0.0001*			
=<5 cm	161	16				
>5; =<10 cm	131	38				
>10 cm	58	33				
**Mitotic Count (50HPFs)^#^**			<0.0001*			
0-5	249	33				
6-10	43	14				
>10	58	40				
**NCCN Guideline**			<0.0001*			<0.0001*
None/Very low	88	3		1	-	
Low	100	10		3.436	2.967-52.632	
Moderate	65	15		3.460	1.601-7.463	
High	97	59		12.658	1.736-6.803	
**Mutation Type**			0.0005*			0.061
Favorable type	106	22		1	-	
Unfavorable type	107	45		1.668	0.977-2.845	
**MGLL expression^#^**			<0.0001*			0.031*
Low Exp.	175	28		1	-	
High Exp.	175	59		1.869	1.058-3.300	

NCCN, National Comprehensive Cancer Network; MGLL, monoglyceride lipase; #, Tumor size and mitotic activity were not introduced in multivariate analysis, since these two parameters were component factors of NCCN guidelines; *, Statistically significant. HR, hazard ratio.

When adopting only the NCCN guidelines in the multivariate analysis for DFS (Table 2), *MGLL* overexpression (*p* = 0.031, hazard ratio [HR] = 1.869) remained an independent prognosticator of poorer outcomes, as did higher GIST risk (*p* < 0.0001) and the presence of epithelioid histology (*p* = 0.001). However, the GISTs harboring mutated *KIT/PDGFRA* of unfavorable genotypes exhibited only a trend toward independent prediction of poorer DFS (*p* = 0.061). We further examined the effect of the NIH scheme (Supplementary Table 3) on the multivariate survival analyses regarding the independence of *MGLL* overexpression on DFS, which demonstrated generally similar results and statistical power to those defined using the NCCN scheme. *MGLL* overexpression (*p* = 0.008, HR = 2.116) again remained the independent prognosticator of poorer outcomes, as did higher NIH risk levels (*p* < 0.001), the presence of epithelioid histology (*p* = 0.003), and unfavorable genotypes (*p* = 0.034). Regarding OS, *MGLL* overexpression still independently predicted adverse events (Supplementary Tables 4 and 5), together with increased risk levels and unfavorable genotypes (coanalyzed using the NCCN guidelines: *p* = 0.032, HR = 2.024; coanalyzed using the NIH scheme: *p* = 0.034, HR = 1.984). Taken together, *MGLL* overexpression and higher risk levels represent independent poor prognosticators, regardless of the risk criteria and endpoints being analyzed.

## DISCUSSION

Among the deregulated metabolic events in carcinogenesis, increasing attention is being focused on the development of lipogenic phenotypes, which lead to tumor growth in various cancer types by providing energy substrates, building blocks for lipid rafts, and oncogenic lipid products [11-13]. In human cancers, oncogenic lipogenesis is prototypically exemplified through increased fatty acid synthase levels, resulting in enhanced de novo fatty acid synthesis and poor prognosis [12]. Fatty acid synthase, predominantly overexpressed in high-risk and metastatic GISTs, has a proproliferative oncogenic attribute through the positive regulation of cyclin A1. In GISTs, we recently reported the negative prognostic effect of aberrantly increased levels of alpha-methylacyl-CoA racemase (AMACR), which functions as a gatekeeper for fueling the β-oxidation of branched-chain fatty acids [8]. Partly driven by gene amplification, the overexpressed AMACR promotes cell proliferation and represents an exploitable target of AMACR chemical inactivators [8]. In this series, we began with transcriptomic reappraisal of aberrantly expressed genes regulating lipid metabolism and systematically validated the clinical relevance of increased MGLL and *MGLL* mRNA levels in GISTs.

In normal adipocytes, MGLL serves as a lipolytic enzyme with serine-hydrolyzing capacity and enables the liberation of free fatty acids by catalyzing the final step of lipolysis to supply fuel for energy consumption [14, 17]. The current knowledge regarding the regulation and biological functions of MGLL in neoplastic diseases remains limited, with the exact role of MGLL in various aspects of cancer biological processes being rarely characterized [13-15]. To complement the swift incorporation of nascent fatty acids into the cellular lipid stores, a molecular mechanism responsible for lipolysis is probably required for liberating fatty acid moieties from this oil depot for fulfilling the requirements of rapidly proliferating cancers [14]. Nomura et al. reported that aggressive cancer lines of various cellular lineages and primary ovarian carcinomas and melanomas may hijack overexpressed MGLL to drive tumorigenesis by remodeling fatty acids to yield a signaling network enriched with pro-oncogenic lipid metabolites such as lipophosphatidic acid and prostaglandins [14]. By contrast, the reduced expression or even absence of MGLL has been reported in various common carcinomas, with MGLL proposed as a potential tumor suppressor [18, 19] because of its growth-suppressive effect on cell colony formation [19]. Notably, contradictory results regarding the oncogenic versus tumor suppressive functions of MGLL have been reported for the same tumor type, such as colorectal cancers [19-21]. These contentions on the functional role of MGLL suggest that its complex disparity in cancer biology may be tumor context-dependent.

Given the strong positive association between *MGLL* mRNA and immunoexpression levels, increased MGLL levels were attributable to the increased mRNA levels in GISTs, at least in part. Compared with the adjacent nontumoral tissue, the *MGLL* mRNA abundance was significantly higher in the entire GIST group, and it increased stepwise in parallel with the increase in the risk. Furthermore, MGLL overexpression was more frequent in GISTs characterized by the nongastric location, increased tumor size and mitosis, and higher risk levels defined using both the NIH and NCCN schemes. Regardless of whichever risk scheme being introduced, MGLL overexpression was notably identified as an independent poor prognosticator of DFS and OS in the large TMA cohort of GISTs, with an approximately 2-fold increased risk of adverse outcome in multivariate analysis. Taken together, these features clearly indicate that MGLL represents an oncogenic lipid-metabolizing enzyme that confers growth advantages to and aggravates the progression of GISTs.

The concomitantly increased *MGLL* mRNA and MGLL levels in GISTs imply transcriptional control as a mechanism regulating *MGLL* expression, which appears inferable from the transcriptional derepression of *MGLL* promoter through the loss of tumor-suppressive PRDM5 transcription factor in intestinal carcinogenesis [19]. However, the regulation of *MGLL* expression may operate at multiple levels. For instance, MGLL was reported as a potential tumor suppressor in hepatocellular carcinomas, associated with its loss of protein expression, which was posttranslationally dictated by SND1-promoted ubiquitination for proteasome-mediated proteolysis [18]. As revealed in the datasets of cancer genomic projects [22], *MGLL* amplification is common in various tumor types such as prostatic carcinomas with neuroendocrine phenotypes [23] and pancreatic ductal adenocarcinomas [24]. Further understanding of the mechanisms underlying deregulated lipid metabolism may provide novel therapeutic strategies for imatinib-refractory GISTs, for which MGLL may represent a promising target metabolic driver in light of the rapid emergence of several novel MGLL-targeting chemical inhibitors [17, 25].

In summary, MGLL was substantiated as a critical lipid-metabolizing enzyme contributing to aggressiveness in GISTs, given its risk increment-associated mRNA upregulation and protein overexpression. *MGLL* overexpression is associated with adverse clincopathological factors and is independently predictive of unfavorable prognosis, suggesting its causative role in conferring aggressive phenotypes to primary localized, imatinib-naïve GISTs. However, the expression status of *MGLL* is associated with neither *KIT/PDGFRA* genotypes nor imatinib resistance or sensitivity. Future studies may further elucidate the molecular underpinning of *MGLL* overexpression in GISTs and the therapeutic relevance of MGLL inhibitors to facilitate the development of alternative targeted therapy for imatinib-resistant GISTs with high-risk aggressiveness.

## MATERIALS AND METHODS

### Reappraisal of published transcriptomic datasets

Focusing on driver(s) deregulated in lipid metabolism, we reappraised transcriptomic dataset of imatinib-naïve gastric and intestinal stromal tumors at various risk levels to search for aberrantly expressed genes critical in tumor progression. These samples were deposited in Gene Expression Omnibus (GSE8167) and profiled for global mRNA expression by using GeneChip Human Genome U133 Plus 2.0 arrays. The raw CEL files were imported into the Nexus Expression 3 software (BioDiscovery Hawthorne, CA, USA) to analyze all probe sets without preselection or filtering. Unsupervised comparative analysis was performed to identify significant genes differentially expressed between the high-risk and non-high-risk samples, with special attention paid to the lipid metabolic process in Gene Ontology (GO: 0006629). The ranking in the expression fold change (at least >1-fold in the log_2_-transformed ratio) and the power of statistical significance (*p* ≤ 0.0001 according to the Student *t* test) were considered for prioritizing potential candidate genes for further validation.

### Tumor cohorts

The institutional review board of Chang Gung Memorial Hospital approved this study (102-3911B). To validate the transcriptomic reappraisal results, we exploited the first cohort of 86 primary localized GISTs with formalin-fixed tissues for assessing *MGLL* mRNA expression level by using QuantiGene assays and MGLL protein immunoexpression on whole sections from 70 cases with informative data of *MGLL* mRNA quantitation (cohort 1 in Supplementary Figure 1). The second cohort comprised 370 primary tumors resected before 2009 (cohort 2 in Supplementary Figure 1), from which triplicate representative cores for each case had been assembled into TMAs used in a previous publication [8]. TMA sections were recut for MGLL immunostaining, yielding 350 informative cases, including 213 successfully determined for *KIT*/*PDGFRA* genotypes as described previously [8]. All cases were imatinib-naïve before disease relapse in both cohorts, the clinicopathological characteristics of which are listed in Table 1 and Supplementary Table 1.

### QuantiGene branched-chain DNA assay

This novel assay employed a sandwich nucleic acid hybridization technique to quantitatively measure the relative mRNA abundance of housekeeping and target transcripts in tissue homogenates obtained from formalin-fixed tumor tissues [26]. In brief, custom probes specifically targeting the *MGLL* transcript were designed for detection through the QuantiGene Multiplex 2.0 assay system (Affymetrix/Panomics Inc., Santa Clara, CA), according to the manufacturer's instructions. Oligonucleotides of the probe set were mixed with the lysed paraffin sections, and the mixture was then added to a 96-well plate coated with capture probe oligonucleotide. Target RNA was captured during overnight incubation at 55°C. Unbound material was removed by three-run washes with 300 μL of wash buffer, followed by the hybridization of DNA amplifier molecules and three additional washes after incubation every time. After the final wash, the dioxetane alkaline phosphatase substrate Lumiphos Plus (Lumingen Inc., Southfield, MI, USA) was added to the reaction wells for detection using a Luminex 100 microplate luminometer (Luminex, Austin, TX, USA). The detected readout of *MGLL* mRNA abundance was further normalized through the expression level of reference glyceraldehyde-3-phosphate dehydrogenase transcript.

### Cell culture

GIST882, GIST48, and GIST430 cell lines were kindly provided by Professor Jonathan Fletcher and cultured by following published methods [8, 16]. In brief, cell lines were maintained in Iscove's modified Dulbecco's medium (Invitrogen, Carlsbad, CA, USA) supplemented with 15% fetal bovine serum (FBS), 100 U/mL penicillin/streptomycin, and 4 mM L-glutamine (Invitrogen) at 37°C in 5% CO_2_. GIST882 was established from an untreated GIST with an imatinib-sensitive K642E mutation in *KIT* exon13. GIST48 and GIST430 were derived from progressing GISTs on imatinib therapy. GIST48 harbored primary homozygous V560D mutation in *KIT* exon11 and secondary heterozygous D820A mutation in *KIT* exon17. GIST430 exhibited primary heterozygous exon 11 in-frame deletion and secondary heterozygous exon13 missense mutation. Primary HCSMCs (ScienCell, Carlsbad, CA, USA) were cultured at 37°C in smooth muscle medium containing 500 mL of basal medium, 10 mL of FBS, 5 mL of growth supplement, and 5 mL of penicillin–streptomycin solution until 90% confluence.

### Western blot analysis

To evaluate the endogenous MGLL expression, equal amounts of total protein (25 μg) extracted from GIST cell lines and primary HCSMCs were separated on 4%–12% gradient NuPAGE gels (Invitrogen), transferred onto PVDF membranes (Amersham, Arlington Heights, IL, USA), and blocked with 5% skim milk in Tris-buffered saline with Tween 20 at room temperature for 1 h. The membranes were then probed with antibodies against MGLL (1:1000, Epitomics, Burlingame, CA, USA) and GADPH (1:3000, Chemicon, Temecula, CA, USA) as the loading control. After incubation with the secondary antibody for 1.5 h, MGLL protein was visualized using an enhanced chemiluminescence system (Amersham) and semiquantitatively measured through densitometry.

### Immunohistochemistry

Whole blocks and TMA sections were microwave-heated to retrieve tissue antigen and incubated with the primary antibody against MGLL (1:100; Epitomics), followed by detection with ChemMate EnVision kit (Dako, Glostrup, Denmark). Blinded to patient outcomes and molecular testing results, one pathologists (I.C.C.) independently assessed cytoplasmic MGLL expression through H-scoring [27], defined by the equation Σ*P_i_* (*i* + 1), where *i* is the intensity of stained tumor cells (0–3+) and *Pi* is the percentage of stained tumor cells (0%–100%). Specifically for the TMA cohort, MGLL overexpression was defined for cases when their means of triplicate H-scores were higher the median value of the 350 informative cases.

### *KIT/PDGFRA* mutation analysis

The methods of direct sequencing of *KIT* exon 11 and denatured high-performance liquid chromatography screening for *KIT* exons 9, 13, and 17 and *PDGFRA* exons 12 and 18 with confirmatory sequencing have been described previously [8, 28].

### Statistical analysis

The Mann–Whitney U test was performed on the full-sectioned samples to determine the difference in *MGLL* mRNA abundance between adjacent normal tissue and GISTs and between the high-risk and non-high-risk groups. Pearson correlation analysis was used to evaluate the association between *MGLL* mRNA abundance and MGLL immunoexpression. In the TMA validation set, we evaluated the associations of MGLL immunoexpression with clinicopathological factors by using the Chi-square and Wilcoxon rank-sum tests for categorical and continuous variables, respectively. Follow-up data were available for 350 cases as of April 2009 (median, 49.9 months; range, 1–247 months). The endpoints were DFS and OS. *KIT/PDGFRA* genotypes were dichotomized into two groups according to prognosis, as reported previously [8, 28]. In brief, the favorable genotypes included *PDGFRA* mutation involving exons 12 or 18, 3′ tandem insertion of *KIT* exon 11 with or without point mutation, and a single point mutation of *KIT* exon 11. The unfavorable genotypes were Ala502-Tyr503 insertion of *KIT* exon 9, wild-type for both *KIT* and *PDGFRA*, and 5′ deletion of *KIT* exon 11 with or without a point mutation. We used the log-rank test to compare univariate prognostic analyses. Significant prognosticators with univariate *p* < 0.05 were generally included in the multivariate Cox regression analysis. As component factors of the NIH risk scheme and NCCN guidelines, tumor size and mitotic activity were not introduced in the multivariate comparisons.

## SUPPLEMENTARY MATERIALS FIGURES AND TABLES





## Abbreviations

GIST: gastrointestinal stromal tumor
*KIT*: KIT protooncogene receptor tyrosine kinase
*MGLL*: monoglyceride lipase
NIH: National Institutes of Health
NCCN: National Comprehensive Cancer Network
*PDGFRA*: platelet-derived growth factor receptor alpha.

## References

[1] Corless CL, Fletcher JA, Heinrich MC (2004). Biology of gastrointestinal stromal tumors. J Clin Oncol.

[2] Antonescu CR, Besmer P, Guo T, Arkun K, Hom G, Koryotowski B, Leversha MA, Jeffrey PD, Desantis D, Singer S, Brennan MF, Maki RG, DeMatteo RP (2005). Acquired resistance to imatinib in gastrointestinal stromal tumor occurs through secondary gene mutation. Clin Cancer Res.

[3] Antonescu CR, Sommer G, Sarran L, Tschernyavsky SJ, Riedel E, Woodruff JM, Robson M, Maki R, Brennan MF, Ladanyi M, DeMatteo RP, Besmer P (2003). Association of KIT exon 9 mutations with nongastric primary site and aggressive behavior: KIT mutation analysis and clinical correlates of 120 gastrointestinal stromal tumors. Clin Cancer Res.

[4] Heinrich MC, Corless CL, Blanke CD, Demetri GD, Joensuu H, Roberts PJ, Eisenberg BL, von Mehren M, Fletcher CD, Sandau K, McDougall K, Ou WB, Chen CJ (2006). Molecular correlates of imatinib resistance in gastrointestinal stromal tumors. J Clin Oncol.

[5] Martín J, Poveda A, Llombart-Bosch A, Ramos R, López-Guerrero JA, García del Muro J, Maurel J, Calabuig S, Gutierrez A, González de Sande JL, Martínez J, De Juan A, Laínez N (2005). Deletions affecting codons 557-558 of the c-KIT gene indicate a poor prognosis in patients with completely resected gastrointestinal stromal tumors: a study by the Spanish Group for Sarcoma Research (GEIS). J Clin Oncol.

[6] Bachet JB, Hostein I, Le Cesne A, Brahimi S, Beauchet A, Tabone-Eglinger S, Subra F, Bui B, Duffaud F, Terrier P, Coindre JM, Blay JY, Emile JF (2009). Prognosis and predictive value of KIT exon 11 deletion in GISTs. Br J Cancer.

[7] Joensuu H, Rutkowski P, Nishida T, Steigen SE, Brabec P, Plank L, Nilsson B, Braconi C, Bordoni A, Magnusson MK, Sufliarsky J, Federico M, Jonasson JG (2015). KIT and PDGFRA mutations and the risk of GI stromal tumor recurrence. J Clin Oncol.

[8] Li CF, Chen LT, Lan J, Chou FF, Lin CY, Chen YY, Chen TJ, Li SH, Yu SC, Fang FM, Tai HC, Huang HY (2014). AMACR amplification and overexpression in primary imatinib-naive gastrointestinal stromal tumors: a driver of cell proliferation indicating adverse prognosis. Oncotarget.

[9] Demetri GD, von Mehren M, Antonescu CR, DeMatteo RP, Ganjoo KN, Maki RG, Pisters PW, Raut CP, Riedel RF, Schuetze S, Sundar HM, Trent JC, Wayne JD (2010). NCCN Task Force report: update on the management of patients with gastrointestinal stromal tumors. J Natl Compr Canc Netw.

[10] Fletcher CD, Berman JJ, Corless C, Gorstein F, Lasota J, Longley BJ, Miettinen M, O'Leary TJ, Remotti H, Rubin BP, Shmookler B, Sobin LH, Weiss SW (2002). Diagnosis of gastrointestinal stromal tumors: A consensus approach. Hum Pathol.

[11] Cairns RA, Harris IS, Mak TW (2011). Regulation of cancer cell metabolism. Nat Rev Cancer.

[12] Flavin R, Peluso S, Nguyen PL, Loda M (2010). Fatty acid synthase as a potential therapeutic target in cancer. Future Oncol.

[13] Liu R, Huang Y (2014). Lipid Signaling in Tumorigenesis. Mol Cell Pharmacol.

[14] Nomura DK, Long JZ, Niessen S, Hoover HS, Ng SW, Cravatt BF (2010). Monoacylglycerol lipase regulates a fatty acid network that promotes cancer pathogenesis. Cell.

[15] Nomura DK, Lombardi DP, Chang JW, Niessen S, Ward AM, Long JZ, Hoover HH, Cravatt BF (2011). Monoacylglycerol lipase exerts dual control over endocannabinoid and fatty acid pathways to support prostate cancer. Chem Biol.

[16] Rossi S, Ou W, Tang D, Bhattacharya N, Dei Tos AP, Fletcher JA, Loda M (2006). Gastrointestinal stromal tumors overexpress fatty acid synthase. J Pathol.

[17] Scalvini L, Piomelli D, Mor M (2016). Monoglyceride lipase: Structure and inhibitors. Chem Phys Lipids.

[18] Rajasekaran D, Jariwala N, Mendoza RG, Robertson CL, Akiel MA, Dozmorov M, Fisher PB, Sarkar D (2016). Staphylococcal nuclease and tudor domain containing 1 (SND1) promotes hepatocarcinogenesis by inhibiting monoglyceride lipase (MGLL). J Biol Chem.

[19] Sun H, Jiang L, Luo X, Jin W, He Q, An J, Lui K, Shi J, Rong R, Su W, Lucchesi C, Liu Y, Sheikh MS (2013). Potential tumor-suppressive role of monoglyceride lipase in human colorectal cancer. Oncogene.

[20] Ye L, Zhang B, Seviour EG, Tao KX, Liu XH, Ling Y, Chen JY, Wang GB (2011). Monoacylglycerol lipase (MAGL) knockdown inhibits tumor cells growth in colorectal cancer. Cancer Lett.

[21] Galli GG, Multhaupt HA, Carrara M, de Lichtenberg KH, Christensen IB, Linnemann D, Santoni-Rugiu E, Calogero RA, Lund AH (2014). Prdm5 suppresses Apc(Min)-driven intestinal adenomas and regulates monoacylglycerol lipase expression. Oncogene.

[22] Cerami E, Gao J, Dogrusoz U, Gross BE, Sumer SO, Aksoy BA, Jacobsen A, Byrne CJ, Heuer ML, Larsson E, Antipin Y, Reva B, Goldberg AP (2012). The cBio cancer genomics portal: an open platform for exploring multidimensional cancer genomics data. Cancer Discov.

[23] Beltran H, Prandi D, Mosquera JM, Benelli M, Puca L, Cyrta J, Marotz C, Giannopoulou E, Chakravarthi BV, Varambally S, Tomlins SA, Nanus DM, Tagawa ST (2016). Divergent clonal evolution of castration-resistant neuroendocrine prostate cancer. Nat Med.

[24] Witkiewicz AK, McMillan EA, Balaji U, Baek G, Lin WC, Mansour J, Mollaee M, Wagner KU, Koduru P, Yopp A, Choti MA, Yeo CJ, McCue P (2015). Whole-exome sequencing of pancreatic cancer defines genetic diversity and therapeutic targets. Nat Commun.

[25] Tuccinardi T, Granchi C, Rizzolio F, Caligiuri I, Battistello V, Toffoli G, Minutolo F, Macchia M, Martinelli A (2014). Identification and characterization of a new reversible MAGL inhibitor. Bioorg Med Chem.

[26] Knudsen BS, Allen AN, McLerran DF, Vessella RL, Karademos J, Davies JE, Maqsodi B, McMaster GK, Kristal AR (2008). Evaluation of the branched-chain DNA assay for measurement of RNA in formalin-fixed tissues. J Mol Diagn.

[27] Ma LJ, Lee SW, Lin LC, Chen TJ, Chang IW, Hsu HP, Chang KY, Huang HY, Li CF (2014). Fibronectin overexpression is associated with latent membrane protein 1 expression and has independent prognostic value for nasopharyngeal carcinoma. Tumor Biol.

[28] Li CF, Huang WW, Wu JM, Yu SC, Hu TH, Uen YH, Tian YF, Lin CN, Lu D, Fang FM, Huang HY (2008). Heat shock protein 90 overexpression independently predicts inferior disease-free survival with differential expression of the alpha and beta isoforms in gastrointestinal stromal tumors. Clin Cancer Res.

