# Supplementary material for: Transcriptomic reappraisal identifies MGLL overexpression as an unfavorable prognosticator in primary gastrointestinal stromal tumors
Source: Oncotarget. 2016 Jun 27;7(31):49986–97. doi: 10.18632/oncotarget.10304 (PMC5226563; doi:10.18632/oncotarget.10304)
Supplement: Supplementary file 1 [file oncotarget-07-49986-s001.pdf]

Transcriptomic reappraisal identifies *MGLL* overexpression as an unfavorable prognosticator in primary gastrointestinal stromal tumors

SUPPLEMENTARY FIGURE AND TABLES

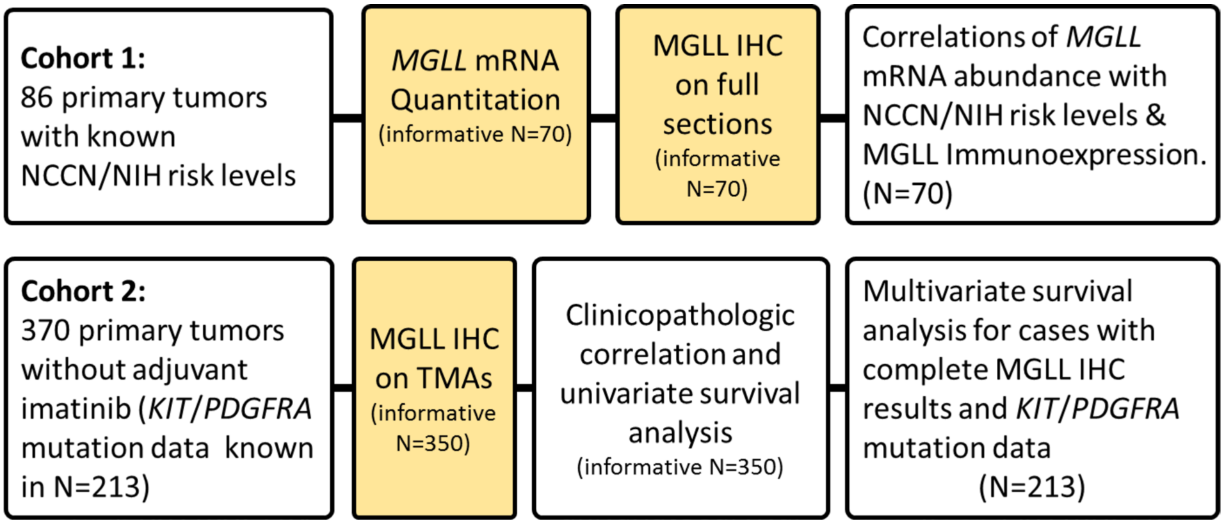

Supplementary Figure S1: Consort diagram of two independent cohorts for validating the clinical relevance of *MGLL* mRNA abundance and its protein expression, respectively.

Supplementary Table S1: Clinicopathological parameters of 70 cases informative for *MGLL* mRNA and protein expression levels using sections from whole tissue blocks

**Sex**	
Male	31
Female	39
**Age (years)**	61.0+/-10.95
**Location**	
Gastric	49
Non-gastric	21
**Histologic Type**	
Spindle	57
Epithelioid & Mixed	13
**Tumor Size (cm)**	5.4+/-3.33
**Mitotic Count (50 HPFs)**	6.3+/-11.31
**NIH Risk**	
Non-high risk	50 (Low/Very low risk, 21; Intermediate risk, 29)
High risk	20
**NCCN Guideline**	
Non-high risk	50 (None/Very low risk, 11; Low risk, 25; Moderate risk, 14)
High risk	20
**Mutation Type**	
Favorable Type	33
Unfavorable Type	37

MGLL, monoglyceride lipase; HPFs, high power fields; NIH, National Institutes of Health; NCCN, National Comprehensive Cancer Network.

Supplementary Table S2: Summary of differentially expressed genes associated with lipid catabolic process in the transcriptome of GIST selected by fold change >+/-1 and p≤0.0001

See Supplementary File 1

Supplementary Table S3: Univariate and multivariate disease-free survival analyses according to MGLL expression status, NIH scheme, and other prognostic factors

Parameter	Univariate analysis			Multivariate analysis		
	No. Case	No. Event	p-value	HR	95% CI	p-value
**Sex**			0.4667			
Male	177	43				
Female	173	44				
**Age (years)**			0.0584			
<70	259	59				
>=70	91	28				
**Location**			**0.0023***			0.253
Gastric	211	40		1	-	
Non-gastric	139	47		1.338	0.812-2.203	
**Histologic Type**			**<0.0001***			**0.003***
Spindle	266	51		1	-	
Mixed/Epithelioid	84	36		2.134	1.297-3.510	
**Tumor Size (cm)#**			**<0.0001***			
=<5 cm	161	16				
>5; =<10 cm	131	38				
>10 cm	58	33				
**Mitotic Count (50HPFs)#**			**<0.0001***			
0-5	249	33				
6-10	43	14				
>10	58	40				
**NIH Consensus scheme**			**<0.0001***			**<0.001***
Very low/Low	127	6		1	-	
Intermediate	110	17		1.597	0.561-4.547	
High	113	64		6.926	2.707-17.724	
**Mutation Type**			**0.0005***			**0.034***
Favorable type	106	22		1	-	
Unfavorable type	107	45		1.780	1.045-3.031	
**MGLL expression#**			**<0.0001***			**0.008***
Low Exp.	175	28		1	-	
High Exp.	175	59		2.116	1.212-3.694	

#, Tumor size and mitotic activity were not introduced in multivariate analysis, since these two parameters were component factors of NIH (National Institutes of Health) risk scheme; *, Statistically significant. HR, hazard ratio; MGLL, monoglyceride lipase.

Supplementary Table S4: Univariate and multivariate overall survival analyses according to MGLL expression status, NCCN guidelines, and other prognostic factors

Parameter	Univariate analysis			Multivariate analysis		
	No. Case	No. Event	p-value	HR	95% CI	p-value
**Sex**			0.2274			
Male	177	31				
Female	173	38				
**Age (years)**			**0.0048***			
<70	259	43		1	-	0.108
>=70	91	26		1.623	0.899-2.932	
**Location**			0.0807			
Gastric	211	35				
Non-gastric	139	34				
**Histologic Type**			**0.0020***			0.575
Spindle	266	44		1	-	
Mixed/Epithelioid	84	25		1.190	0.647-2.189	
**Tumor Size (cm)#**			**<0.0001***			
=<5 cm	161	20				
>5; =<10 cm	131	24				
>10 cm	58	25				
**Mitotic Count (50HPFs)#**			**<0.0001***			
0-5	249	34				
6-10	43	10				
>10	58	25				
**NCCN Guideline**			**<0.0001***			**0.018***
None/Very low	88	7		1	-	
Low	100	13		2.222	0.799-6.173	
Moderate	65	10		2.625	1.072-6.410	
High	97	39		3.311	1.403-7.813	
**Mutation Type**			**0.0001***			**0.007***
Favorable type	106	13		1	-	
Unfavorable type	107	37		2.458	1.277-4.731	
**MGLL expression#**			**0.0007***			**0.032***
Low Exp.	175	22		1	-	
High Exp.	175	47		2.024	1.061-3.863	

NCCN, National Comprehensive Cancer Network. MGLL, monoglyceride lipase. #, Tumor size and mitotic activity were not introduced in multivariate analysis, since these two parameters were component factors of NCCN guidelines; *, Statistically significant. HR, hazard ratio.

Supplementary Table S5: Univariate and multivariate overall survival analyses according to MGLL expression status, NIH scheme, and other prognostic factors

Parameter	Univariate analysis			Multivariate analysis		
	No. Case	No. Event	p-value	HR	95% CI	p-value
**Sex**			0.2274			
Male	177	31				
Female	173	38				
**Age (years)**			**0.0048***			0.167
<70	259	43		1	-	
>=70	91	26		1.519	0.840-2.747	
**Location**			0.0807			
Gastric	211	35				
Non-gastric	139	34				
**Histologic Type**			**0.0020***			0.765
Spindle	266	44		1	-	
Mixed/Epithelioid	84	25		1.096	0.600-2.003	
**Tumor Size (cm)#**			**<0.0001***			
=<5 cm	161	20				
>5; =<10 cm	131	24				
>10 cm	58	25				
**Mitotic Count (50HPFs)#**			**<0.0001***			
0-5	249	34				
6-10	43	10				
>10	58	25				
**NIH Consensus scheme**			**<0.0001***			**0.003***
Very low/Low	127	13		1	-	
Intermediate	110	10		1.567	0.546-4.484	
High	113	46		2.742	1.161-6.474	
**Mutation Type**			**0.0001***			**0.004***
Favorable type	106	13		1	-	
Unfavorable type	107	37		2.568	1.349-4.890	
**MGLL expression#**			**0.0007***			**0.034***
Low Exp.	175	22		1	-	
High Exp.	175	47		1.984	1.052-3.743	

NIH, National Institutes of Health. MGLL, monoglyceride lipase. #, Tumor size and mitotic activity were not introduced in multivariate analysis, since these two parameters were component factors of NIH risk scheme; *, Statistically significant. HR, hazard ratio.
