# Supplementary material for: Transcriptomic reappraisal identifies MGLL overexpression as an unfavorable prognosticator in primary gastrointestinal stromal tumors
Source: Oncotarget. 2016 Jun 27;7(31):49986–97. doi: 10.18632/oncotarget.10304 (PMC5226563; doi:10.18632/oncotarget.10304)
Supplement: Supplementary file 2 [file oncotarget-07-49986-s002.doc]

**Table-S2 Summary of differentially expressed genes associated with lipid metabolic process in the transcriptome of GIST selected by fold change >+/-1 and p≤0.0001**

| **Probe** | **Comparison log ratio** | **Comparison p-value** | **Gene Symbol** | **Gene Name** | **Biological Process** | **Molecular Function** |
| --- | --- | --- | --- | --- | --- | --- |
| 208791_at | -2.3136 | <0.0001 | ***CLU*** | clusterin | apoptosis, cell death, complement activation, complement activation; classical pathway, immune response, innate immune response, lipid metabolic process |  |
| 208792_s_at | -2.2513 | <0.0001 | ***CLU*** | clusterin | apoptosis, cell death, complement activation, complement activation; classical pathway, immune response, innate immune response, lipid metabolic process |  |
| 218718_at | -1.8081 | <0.0001 | ***PDGFC*** | platelet derived growth factor C | cell proliferation, central nervous system development, lipid metabolic process, regulation of progression through cell cycle | growth factor activity, hydrolase activity, hydrolase activity; acting on ester bonds |
| 220356_at | -1.3961 | <0.0001 | ***CORIN*** | corin; serine peptidase | anatomical structure morphogenesis, blood pressure regulation, lipid metabolic process, proteolysis | hydrolase activity, peptidase activity, scavenger receptor activity, serine-type endopeptidase activity |
| 222043_at | -2.299 | <0.0001 | ***CLU*** | clusterin | apoptosis, cell death, complement activation, complement activation; classical pathway, immune response, innate immune response, lipid metabolic process |  |
| 203895_at | 1.2642 | <0.0001 | ***PLCB4*** | phospholipase C; beta 4 | intracellular signaling cascade, lipid catabolic process, lipid metabolic process, signal transduction | calcium ion binding, hydrolase activity, phosphoinositide phospholipase C activity, phospholipase C activity, protein binding, signal transducer activity |
| 203896_s_at | 1.1827 | 0.0001 | ***PLCB4*** | phospholipase C; beta 4 | intracellular signaling cascade, lipid catabolic process, lipid metabolic process, signal transduction | calcium ion binding, hydrolase activity, phosphoinositide phospholipase C activity, phospholipase C activity, protein binding, signal transducer activity |
| 205404_at | 2.7352 | <0.0001 | ***HSD11B1*** | hydroxysteroid (11-beta) dehydrogenase 1 | lipid metabolic process, lung development, metabolic process, steroid metabolic process | 11-beta-hydroxysteroid dehydrogenase (NADP+) activity, 11-beta-hydroxysteroid dehydrogenase activity, oxidoreductase activity |
| 211026_s_at | 1.3407 | <0.0001 | ***MGLL*** | monoglyceride lipase | aromatic compound metabolic process, inflammatory response, lipid metabolic process | acylglycerol lipase activity, catalytic activity, hydrolase activity, lysophospholipase activity |
| 225102_at | 1.1483 | <0.0001 | ***MGLL*** | monoglyceride lipase | aromatic compound metabolic process, inflammatory response, lipid metabolic process | acylglycerol lipase activity, catalytic activity, hydrolase activity, lysophospholipase activity |
